# Comparative Effectiveness of Acupuncture Versus Non-surgical Modalities for Treating Plantar Fasciitis: A Network Meta-Analysis

**DOI:** 10.7759/cureus.68959

**Published:** 2024-09-08

**Authors:** Indrarajah Asokumaran, Bavithaa Sufina Verasamy, Mohd Idham B Hasan, Danny Kit Chung Wong, Siew Siew Ong, Shih Chau Ng

**Affiliations:** 1 Orthopaedics and Traumatology, Putrajaya Hospital, Putrajaya, MYS; 2 Integrative and Complementary Medicine, International Medical University, Kuala Lumpur, MYS; 3 Otolaryngology and Head and Neck Surgery, Sungai Long Specialist Hospital, Kajang, MYS

**Keywords:** acupuncture therapy, corticosteroid injection, extracorporeal shockwave therapy, network analysis, plantar fasciitis, plantar heel pain, platelet-rich plasma (prp), recalcitrant plantar fasciitis, ultrasound foot

## Abstract

Plantar fasciitis, or plantar heel pain, causes inflammation of the plantar fascia due to various causes, with no clear consensus on the treatment protocol. Standard first-line treatment includes non-steroidal anti-inflammatory drugs and physiotherapy. Second-line treatment prior to surgery includes extracorporeal shockwave therapy (ESWT), ultrasound-guided (USG) therapy, corticosteroid injection (CSI), and platelet-rich plasma (PRP) injection. Recently, the use of acupuncture treatment has been gaining popularity, with increasing published evidence showing its effectiveness in treating plantar fasciitis. The objective of this study was to determine whether acupuncture intervention was a viable alternative treatment method for managing plantar fasciitis when compared to ESWT, USG therapy, CSI, and PRP injection. Data sources from PubMed, Google Scholar, Scopus, Science Direct, and China National Knowledge Infrastructure were reviewed. Clinical trials were searched from their inception over the period of January 2000 to October 2020. A total of 32 relevant papers were included for analysis, totaling 2390 samples. Visual Analog Scale (VAS) scores measuring pain were analyzed in terms of outcome after one and three months of treatment. Each time point was analyzed separately through a network meta-analysis using the frequentist approach. VAS scores for each intervention at baseline and the two-time points (i.e., one and three months) were included in the comprehensive meta-analysis. Then, differences in VAS scores were calculated in R studio (V4.1.2; RStudio: Integrated Development for R, RStudio, Inc., Boston, USA) using the netmeta package. The netmeta package was also used to perform the network meta-analysis and generate corresponding figures. Direct and indirect effects were assessed and visualized through a direct evidence plot and a node-splitting forest plot. Randomized controlled trials (RCTs) and non-RCTs involving treatments of acupuncture, ESWT, USG therapy, CSI, or PRP injection, either in comparison with each other or with a placebo, were included in our review. Our meta-analysis showed that at one month, VAS scores for acupuncture treatment had the highest mean difference (MD) of -1.33 (95% confidence interval (95% CI) = -2.19 to -0.46) compared to placebo, indicating that acupuncture treatment was more effective than other treatment arms when compared to placebo. Analysis at threemonths showed that the highest-ranked treatment was PRP injection, with an MD of -2.67 (95% CI = -6.23 to 0.89). However, the CI for the net effect of all treatments crossed the null effect on the forest plot, indicating no statistically significant difference between the treatment and placebo. Acupuncture treatment should be considered as a second-line treatment for treatment of plantar fasciitis together with other common treatment options such as ESWT, PRP injection, CSI, and USG therapy. Further long-term studies measuring acupuncture treatment outcomes would be beneficial in the future.

## Introduction and background

Plantar fasciitis, a form of inflammation of the plantar fascia, is a common condition, especially affecting working adults with prolonged standing. Presentation usually involves pain and discomfort over the medial aspect of the inferior heel region. Patients with plantar fasciitis often complain of severe excruciating pain upon weight-bearing after rising from their bed in the early morning, with the pain and discomfort eventually subsiding after taking a certain number of steps.

Diagnosis of plantar fasciitis is made by ruling out other pathologies involving heel pain. This condition is a type of overuse injury that is typically associated with intrinsic and extrinsic factors [1]. Extrinsic factors include environmental factors such as poor biomechanics, hard surface ambulation, prolonged weight-bearing, inadequate stretching, and poor footwear. Intrinsic factors are divided into anatomic and biomechanics factors. Anatomic factors include obesity, pes planus, pes cavus, and a tight Achilles tendon, whereas biomechanical factors include overpronation, weak intrinsic muscles of the foot, and weak plantar flexor muscles.

The presence of calcaneal spurs, osseous prolongations located at the medial process of the plantar calcaneal tuberosity, has been previously suggested as an intrinsic factor contributing to plantar fasciitis. However, the presence of heel spur is not diagnostic of plantar fasciitis. In a study of radiographic images involving 1000 patients, only 5.2% with heel spurs had symptoms of plantar fasciitis [2].

Treatment of plantar fasciitis is divided into non-surgical and surgical management. There is a wide range of non-surgical management options available, including rest and activity modification, various stretching techniques, weight-loss therapy, anti-inflammatory medication with shoe wear modification, night splints, and specific physical therapies [3-5]. Physical therapy may include eccentric stretches and deep myofascial massage. More advanced physiotherapy equipment recently used to enhance tissue healing includes ultrasound-guided (USG) therapy and extracorporeal shock wave therapy (ESWT). The use of injections, such as corticosteroid injection (CSI) or platelet-rich plasma (PRP) injection, has gained popularity in managing plantar fasciitis. These modes of treatment are usually compared to other types of conservative treatment or among themselves. Acupuncture treatment has shown promising results in successfully managing plantar fasciitis when other modes have failed. However, there has not been a direct comparison between acupuncture, USG therapy, ESWT, and prolotherapy.

The concept of acupuncture treatment is different from dry needling, where understanding the cause of heel pain in Traditional Chinese Medicine (TCM) theory is essential in the former. Acupuncture uses a meridian reference with an additional Ashi point or trigger point, whereas dry needling only utilizes trigger points.

The objective of this study was to perform a network meta-analysis comparing five modes of treatment for plantar fasciitis: acupuncture, USG therapy, ESWT, CSI, and PRP injection. In a literature search specifically looking at second-line treatment for plantar fasciitis, the results showed that treatments such as USG therapy, ESWT, and CSI were the most frequently studied and compared in randomized controlled trials (RCTs) [6-22]. There were very few studies comparing these treatments to acupuncture treatment for plantar fasciitis. There was especially no literature that used a network meta-analysis method to compare acupuncture intervention against USG therapy, ESWT, or prolotherapy to manage plantar fasciitis. However, there have been meta-analysis studies comparing USG therapy, ESWT, and prolotherapy with dry needling [8,23-25]. This study is the first network meta-analysis using specific direct and indirect methods to compare these interventions. To strengthen the statistical power of the analysis, non-randomized prospective studies were included.

## Review

Materials and methods

Study Eligibility Criteria

Types of patients: This study included adult patients who were ≥18 years of age and diagnosed clinically by a physician to have plantar fasciitis. Studies not clearly stating the diagnosis of plantar fasciitis or related to plantar fasciitis as a diagnosis were not included.

Types of studies: The meta-analysis included RCTs and non-randomized prospective studies. Case reports, case series, reviews, and retrospective studies were excluded.

Types of intervention: The meta-analysis included studies comparing interventions that consisted of acupuncture, USG therapy, ESWT, CSI, and PRP injection as part of treatment. Electro-acupuncture was included, as it falls under acupuncture with tonifying effect.

Primary outcome: Studies were included if their primary outcome measure was the Visual Analog Scale (VAS) score used to indicate pain. In this study, VAS scores were adjusted to a centimeter scale ranging from 0 to 10 cm, where 0 represented painless status, and 10 represented unbearable pain. Short-term outcomes were measured at the one-month and three-month marks. We did not include secondary outcomes because they can vary significantly between studies in terms of definition, measurement methods, and reporting. This variability can introduce additional heterogeneity, complicate the synthesis of results, and potentially reduce the reliability of the findings.

Information Sources and Search Strategy

PubMed, Science Direct, Scopus, Google Scholar, and China National Knowledge Infrastructure (CKNI) database were searched with an established method for randomized and non-randomized trials with similar end results for USG therapy, ESWT, CSI, PRP injection, and acupuncture intervention. The search terminologies included “plantar fasciitis,” “plantar fasciopathy,” “plantar fasciosis,” “plantar heel pain (PHP),” and “acupuncture” or “electro-acupuncture” or “ultrasound” or “USG” or “extracorporeal shock wave therapy” or “ESWT” or “corticosteroid” or “steroid injection” or “platelet rich plasma” or “PRP” and “randomized controlled trial” or “RCT” or “controlled clinical trial” or “non-randomized controlled trial.” The bibliographies of relevant review articles and selected articles were examined for additional potentially relevant trials.

Study Selection

All duplicated literature was removed manually. Titles and abstracts were reviewed by two authors (IA and BV) independently. Studies that did not meet the eligibility criteria were removed. The full texts of the remaining studies were then reviewed by the two authors independently. Studies meeting eligibility were included after this process. If there was disagreement between the two authors regarding any study, it was then reviewed by another author (CS). No automation tools were used.

Data Extraction

Both the above-mentioned authors (IA and BV) independently extracted the following data from eligible studies: first author, year of publication, area, study duration, sample size, age, percentage of gender difference, inclusion/exclusion criteria, detailed intervention in each group, number of patients in each group, follow-up time, and outcomes in each group. If required data were unavailable for a study, its authors were contacted directly.

Study Risk of Bias Assessment

Two authors (IA and BV) independently assessed the risk of bias for the selected studies by using the ROBINS-I (Risk Of Bias In Non-randomised Studies - of Interventions) bias assessment tool (Table 1) [26]. This assessment was further verified and confirmed by the supervising author (CS).

**Table 1:** ROBINS-I bias assessment tool ROBINS-I: Risk Of Bias In Non-randomised Studies - of Interventions

Bias Due to Confounding	Bias in the Selection of Participants in the Study	Bias Due to Classification of Interventions	Bias Due to Deviations From Intended Interventions	Bias Due to Missing Data	Bias in Measurement of Outcomes	Bias in Selection of the Reported Result	Overall risk of Bias	User Notes	Title
Low	Moderate	Moderate	Low	Low	Low	Low	Low	-	Akinoglu et al., 2017 [7]
Moderate	Low	Low	Low	Low	Low	Low	Low	-	Xu et al., 2020 [20]
Moderate	Low	Moderate	Moderate	Low	Low	Low	Low	-	Ho et al., 2021 [23]
Low	Low	Low	Low	Low	Low	Low	Low	-	Abdihakin et al., 2012 [27]
Low	Low	Low	Low	Low	Low	Low	Low	-	Acosta-Olivo et al., 2017 [28]
Moderate	Serious	Moderate	Serious	Low	Moderate	Moderate	Moderate	-	Ashahin et al., 2012 [29]
Low	Low	Low	Low	Low	Low	Low	Low	-	Buchbinder et al., 2002 [30]
Moderate	Low	Low	Low	Low	Low	Low	Low	-	Eslamian et al., 2016 [31]
Low	Low	Low	Low	Low	Low	Low	Low	-	Gerdesmeyer et al., 2008 [32]
Low	Low	Low	Low	Low	Low	Low	Low	-	Haake et al., 2003 [33]
Low	Low	Moderate	Low	Low	Low	Low	Low	-	Hammer et al., 2003 [34]
Low	Low	Low	Low	Low	Low	Low	Low	-	Ibrahim et al., 2010 [35]
Moderate	Low	Low	Low	Moderate	Low	Low	Low	-	Jain et al., 2015 [36]
Low	Low	Low	Low	Low	Low	Low	Low	-	Karagounis et al., 2011 [37]
Moderate	Low	Low	Low	Low	Low	Low	Low	-	Konjen et al., 2015 [38]
Moderate	Moderate	Low	Low	Low	Low	Low	Low	-	Krukowska et al., 2016 [39]
Low	Low	Low	Low	Low	Low	Low	Low	-	Kudo et al., 2006 [40]
Low	Low	Low	Low	Low	Low	Low	Low	-	Kumnerddee et al., 2012 [41]
Low	Low	Low	Low	Low	Low	Low	Low	-	Lai et al., 2018 [42]
Moderate	Moderate	Moderate	Moderate	Moderate	Low	Low	Moderate	-	Mahindra et al., 2016 [43]
Moderate	Low	Low	Low	Low	Low	Low	Low	-	Mardani-Kivi et al., 2015 [44]
Low	Low	Low	Low	Moderate	Low	Low	Low	-	Marks et al., 2008 [45]
Low	Low	Low	Low	Low	Low	Low	Low	-	Omar et al., 2012 [46]
Moderate	Moderate	Moderate	Moderate	Low	Low	Low	Moderate	-	Sahoo et al., 2020 [47]
Moderate	Moderate	Moderate	Moderate	Low	Low	Low	Moderate	-	Say et al., 2014 [48]
Moderate	Moderate	Moderate	Moderate	Low	Low	Low	Moderate|||Low	-	Speed et al., 2003 [49]
Moderate	Serious	Moderate	Low	Low	Low	Low	Moderate	-	Tabrizi et al., 2020 [50]
Low	Low	Low	Low	Low	Low	Low	Low	-	Theodore et al., 2004 [51]
Low	Low	Low	Low	Moderate	Low	Low	Low	-	Tiwari and Bhargava, 2013 [52]
Moderate	Serious	Moderate	Moderate	Moderate	Low	Low	Moderate	-	Wang et al., 2012 [53]
Moderate	-	Moderate	Moderate	Low	Low	Low	Moderate	-	Yucel et al., 2010 [54]
Low	Low	Moderate	Low	Low	Low	Low	Low	-	Yucel et al., 2013 [55]

Statistical Analysis

Network meta-analysis using the frequentist approach was used to compare the direct and indirect effects of different treatment arms on VAS scores. The efficacy of the interventions was assessed by analyzing the change in VAS score from baseline and two-time points: one and three months. Each time point was analyzed separately through a network meta-analysis using the frequentist approach. The data were extracted and cleaned in an Excel file (Microsoft® Corp., Redmond, USA), and the mean difference (MD) between the baseline and the two-time points of each intervention was calculated using a comprehensive meta-analysis. Then, changes in the MDs of the VAS score were calculated in R studio (V4.1.2; RStudio: Integrated Development for R, RStudio, Inc., Boston, USA) using the netmeta package. The netmeta package also was used to perform the network meta-analysis and generate corresponding figures. Direct and indirect effects were assessed and visualized through a direct evidence plot and a node-splitting forest plot. Additionally, a net heat plot was used to assess heterogeneity by comparing both fixed and random effects. After the validity of the network was assessed, a forest plot of the network was used to show the rank and net effect of each intervention in comparison to placebo, and a network graph showed the connections between treatments and a number of studies for each comparison. Moreover, a table containing a matrix of all possible comparisons was produced to check the estimates of each comparison.

Risk of Bias Assessment

Publication bias was assessed using a comparison-adjusted funnel plot. The p-values were calculated with Egger's test, and the publication bias was considered significant when p < 0.05.

Results

Literature Search and Study Selection Results

**Figure 1:**
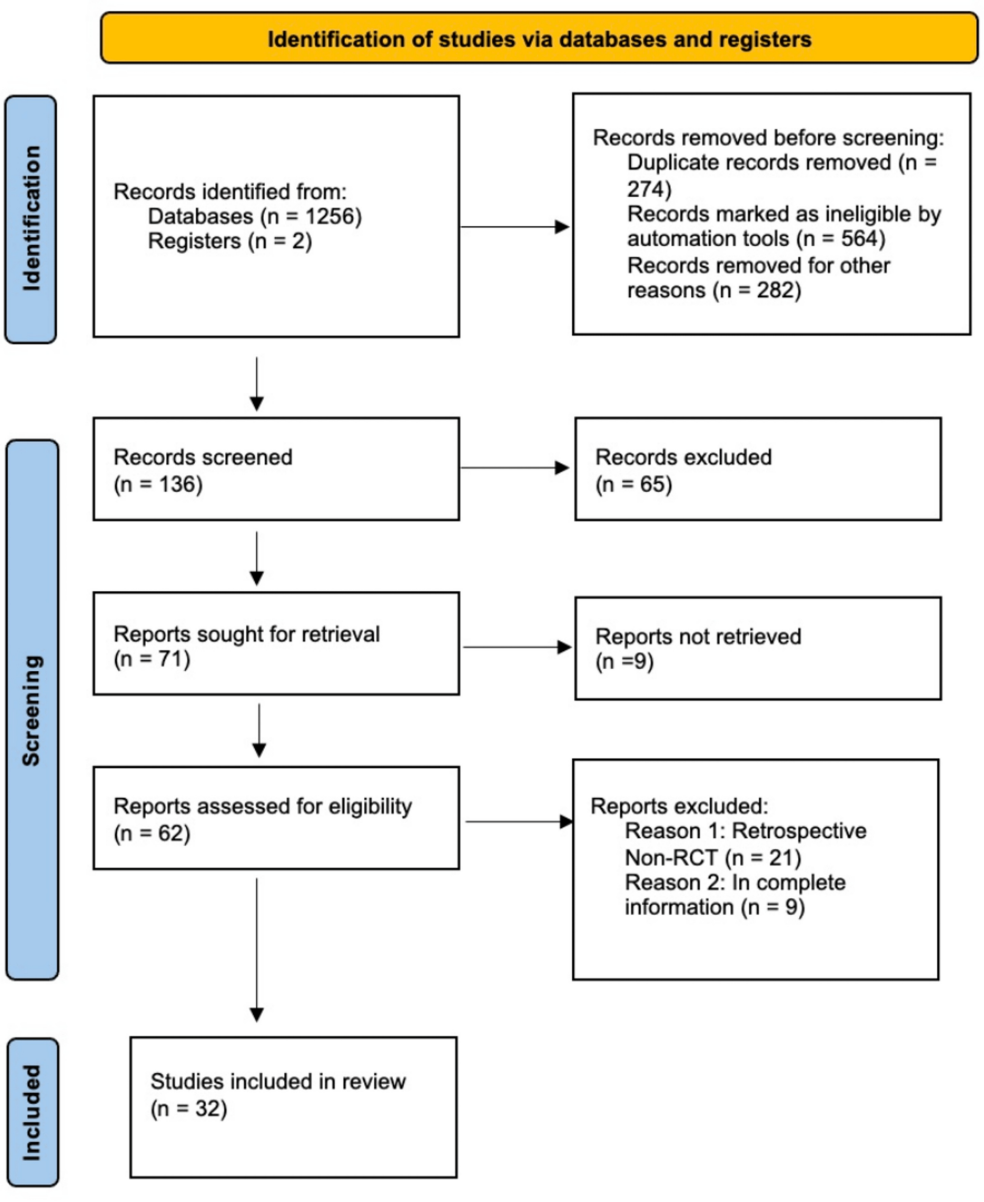
PRISMA flow chart of the study process PRISMA: Preferred Reporting Items for Systemic review and Meta-Analyses; RCT: Randomized controlled trial

A total of 1258 studies were initially searched from PubMed, Scopus, Google Scholar, Science Direct, CKNI, and an additional search from other resources. Out of 1258 studies, 136 studies were screened; others were eliminated initially due to being duplicate studies, systemic reviews, or not meeting the inclusion criteria. Finally, a total of 32 suitable studies were selected for analysis, with a total of 2390 samples from the selected studies. Figure 1 shows a flow diagram of the literature search as recommended in Preferred Reporting Items for Systematic Reviews and Meta-Analyses [26].

Characteristics of the Studies

Characteristics of the studies included in the meta-analysis are shown in Table 2. After the elimination of studies from the search due to eligibility criteria and duplications, we included 32 studies with 2390 samples [7,22,23,27-55]. Out of the 32 studies, one study had three interventions and a placebo, whereas the remaining studies had two interventions. Fifteen studies had placebo comparisons. Among the 32 studies, 16 were double-blinded, five were single-blinded, two were open-label, and the remaining nine trials did not mention their blinding method. Out of the 2390 total samples analyzed, 714 were placebo, 840 were EWST, 473 were CSI, 216 were PRP injection, 94 were acupuncture, and 53 were USG therapy.

**Table 2:** Characteristics of studies ACU: Acupuncture; USG: Ultrasound; ESWT: Extracorporeal shock wave therapy; PRP: Platelet-rich plasma; CS: Corticosteroid injection; T1: Treatment 1; T2: Treatment 2; T3: Treatment 3; NA: Not available; BMI: Body mass index

S. No.	Author, Year	Blinding	Follow-up (months)	T1	Cases	Male/Female	Age (years)	BMI (kg/m²)	T2	Cases	Male/Female	Age (years)	BMI (kg/m²)	T3	Cases	Male/Female	Age (years)	BMI (kg/m²)
1	Akinoglu et al., 2017 [7]	Single	1	ESWT	18	NA	50.0	28.6	USG	18	NA	50.1	28.5	-	-	-	-	-
2	Xu et al., 2020 [22]	NA	6	ESWT	49	13/37	48.5	23.7	CS	47	19/31	47.2	23.1	-	-	-	-	-
3	Ho et al., 2021 [23]	NA	2	ACU	40	6/34	59.4	23.7	PL	40	6/34	60.0	57.7	-	-	-	-	-
4	Abdihakin et al., 2012 [27]	Double	2	CS	47	23/24	41.0	31.7	PL	41	19/22	45.1	31.7	-	-	-	-	-
5	Acosta-Olivo et al., 2017 [28]	Double	4	CS	14	NA	44.8	NA	PRP	14	NA	44.8	NA	-	-	-	-	-
6	Ashahin et al., 2012 [29]	NA	6	CS	30	12/18	46.4	32.77	PRP	30	13/17	45.7	29.6	-	-	-	-	-
7	Buchbinder et al., 2002 [30]	Double	3	ESWT	80	34/46	52.2	29.4	PL	81	33/47	54.2	28.7	-	-	-	-	-
8	Eslamian et al., 2016 [31]	Single	2	ESWT	20	2/18	41.5	NA	CS	20	5/15	42.9	NA	-	-	-	-	-
9	Gerdesmeyer et al., 2008 [32]	Double	12	ESWT	125	NA	52.4	27.2	PL	118	NA	52.0	28.0	-	-	-	-	-
10	Haake et al., 2003 [33]	Double	12	ESWT	127	37/98	53.1	29.4	PL	129	30/106	52.9	29.7	-	-	-	-	-
11	Hammer et al., 2003 [34]	NA	24	ESWT	25	5/19	51	29.6	PL	24	10/13	48	28.7	-	-	-	-	-
12	Ibrahim et al., 2010 [35]	Double	6	ESWT	25	7/18	56.6	NA	PL	25	11/14	49.1	NA	-	-	-	-	-
13	Jain et al., 2015 [36]	NA	12	PRP	23	16/30	55.6	NA	CS	23	6/28	44.7	29.1	-	-	-	-	-
14	Karagounis et al., 2011 [37]	Single	2	ACU	19	NA	36.8	23.21	PL	19	NA	36.8	23.74	-	-	-	-	-
15	Konjen et al., 2015 [38]	Double	6	US	15	2/13	45	26.67	ESWT	15	4/11	45.6	26.03	-	-	-	-	-
16	Krukowska et al., 2016 [39]	Open Label	0.5	USG	20	20/13	51.1	NA	ESWT	27	4/11	51.4	NA	-	-	-	-	-
17	Kudo et al., 2006 [40]	Double	3	ESWT	53	18/40	51.1	NA	PL	52	23/33	48.8	NA	-	-	-	-	-
18	Kumnerddee et al., 2012 [41]	Open Label	1.5	ACU	15	3/12	52.4	25.2	PL	15	0/15	53.8	25.3	-	-	-	-	-
19	Lai et al., 2018 [42]	NA	3	ESWT	47	21/26	54.53	NA	CS	50	22/28	54.58	NA	-	-	-	-	-
20	Mahindra et al., 2016 [43]	Double	3	PRP	25	8/17	30.7	NA	CS	25	12/13	33.9	NA	PL	25	11/14	35.48	NA
21	Mardani-Kivi et al., 2015 [44]	Double	3	ESWT	43	5/29	43.9	30.2	CS	41	6/28	44.7	29.1	-	-	-	-	-
22	Marks et al., 2008 [45]	Double	6	ESWT	16	7/9	52	NA	PL	9	4/5	51.9	NA	-	-	-	-	-
23	Omar et al., 2012 [46]	Double	3	PRP	15	0/15	42.5	NA	CS	15	0/15	44.5	NA	-	-	-	-	-
24	Sahoo et al., 2020 [47]	NA	6	PRP	39	14/25	39.4	NA	CS	38	17/17	38.3	NA	-	-	-	-	
25	Say et al., 2014 [48]	NA	6	CS	25	5/20	47	NA	PRP	25	6/19	48.6	NA	-	-	-	-	
26	Speed et al., 2003 [49]	Double	6	ESWT	46	20/26	51.7	NA	PL	42	17/25	52.5	NA	-	-	-	-	
27	Tabrizi et al., 2020 [50]	Single	24	CS	16	1/15	31.7	32.4	PRP	15	1/14	33.6	33.9	-	-	-	-	
28	Theodore et al., 2004 [51]	Double	12	ESWT	76	14/62	50	NA	PL	74	24/47	53	NA	-	-	-	-	
29	Tiwari and Bhargava, 2013 [52]	Double	6	PRP	30	NA	NA	NA	CS	30	NA	NA	NA	-	-	-	-	
30	Wang et al., 2012 [53]	NA	NA	ACU	20	10/10	47.0	NA	ESWT	20	0/20	40.5	NA	-	-	-	-	
31	Yucel et al., 2010 [54]	Single	1	CS	20	4/16	45.6	30.8	PL	20	4/16	47.4	29.3	-	-	-	-	
32	Yucel et al., 2013 [55]	Double	3	CS	33	5/28	44.7	NA	ESWT	28	13/14	42.9	NA	-	-	-	-	

Change in VAS Score After One Month

**Figure 2:**
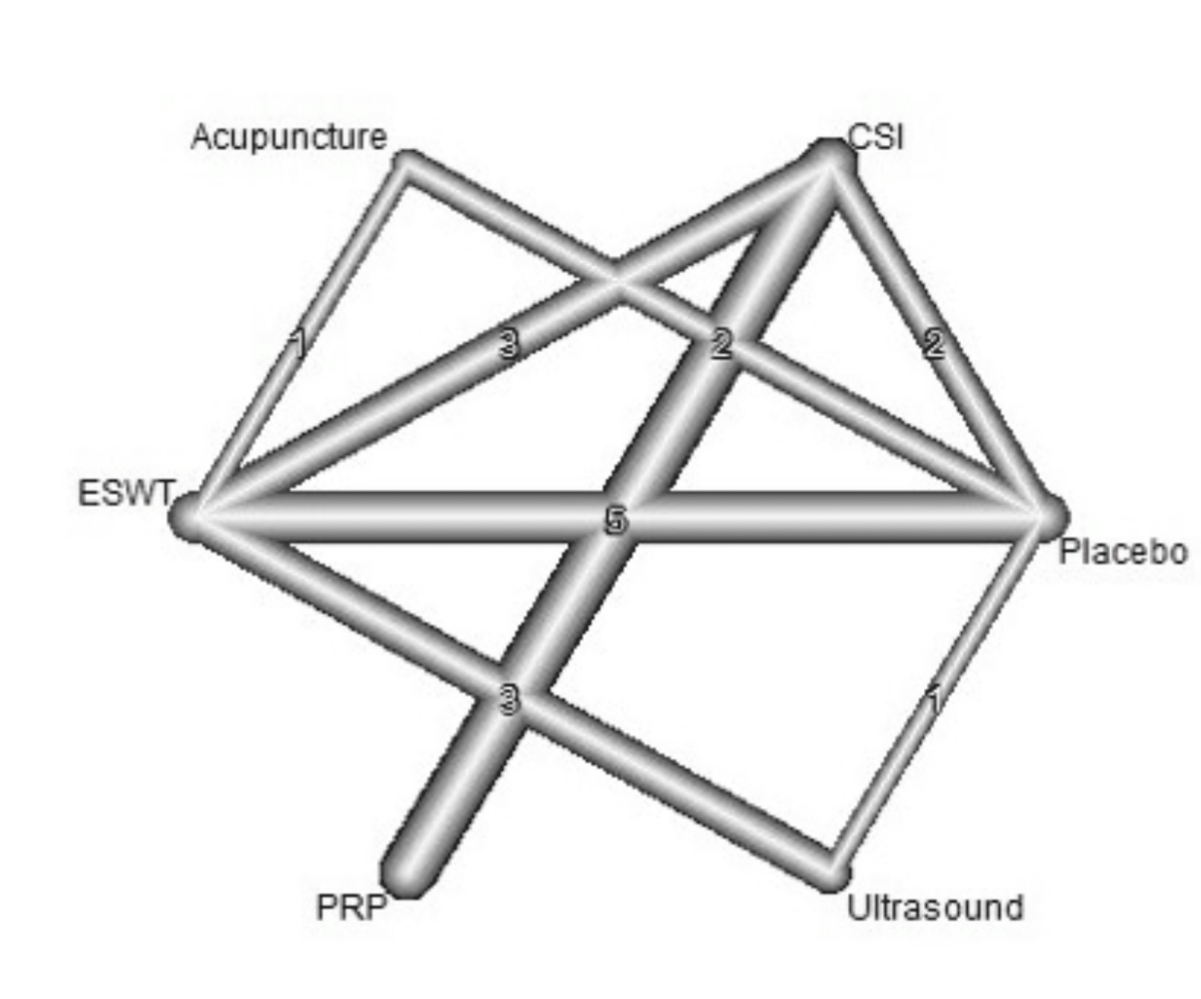
Network graph showing the connections between interventions and number of studies for each comparison for change of VAS score after one month. ESWT: Extracorporeal shock wave therapy; PRP: Platelet-rich plasma; CSI: Corticosteroid injection; VAS: Visual analog scale

**Figure 3:**
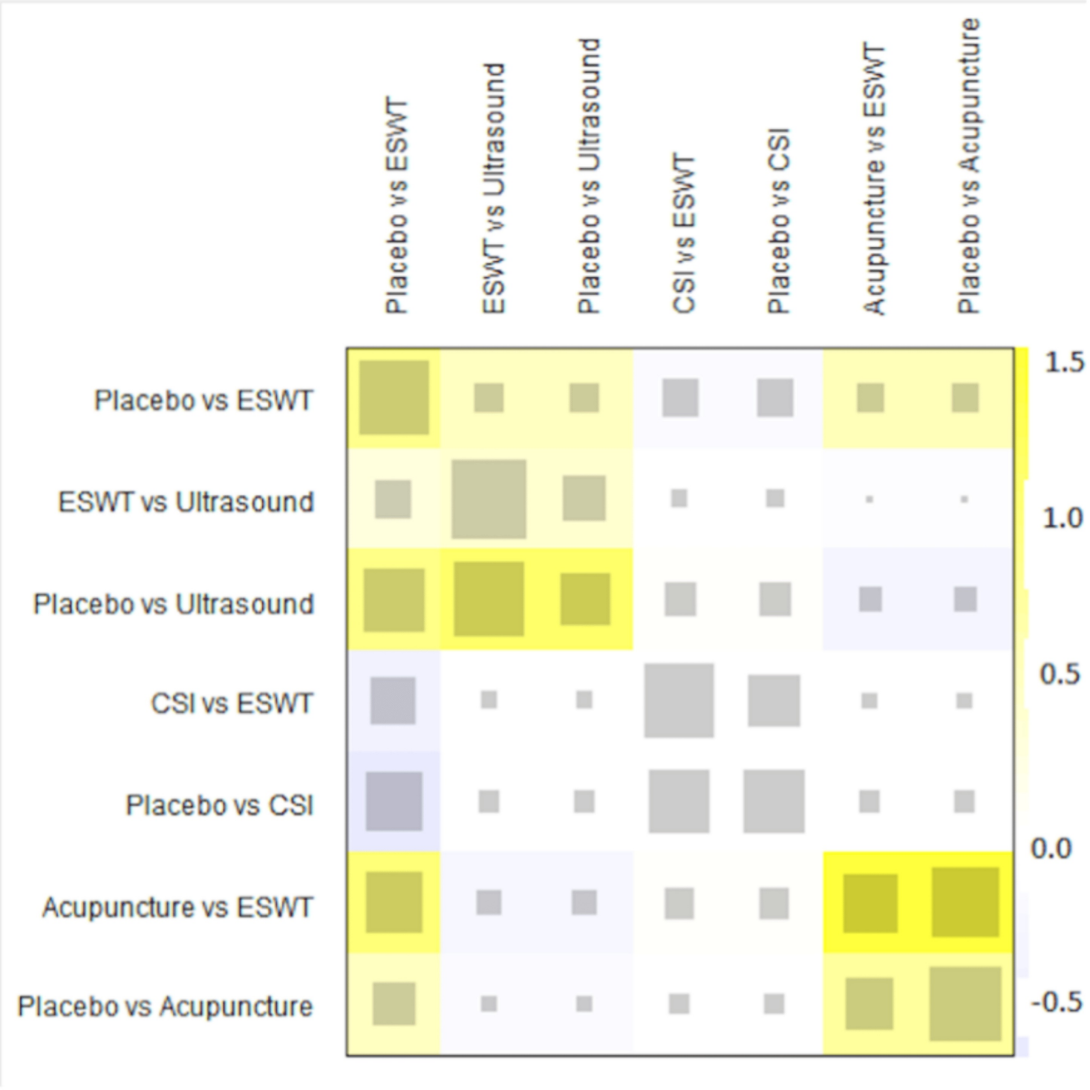
Net heat plot showing the consistency of the network for change of VAS score after one month. ESWT: Extracorporeal shock wave therapy; CSI: Corticosteroid injection; VAS: Visual analog scale

**Figure 4:**
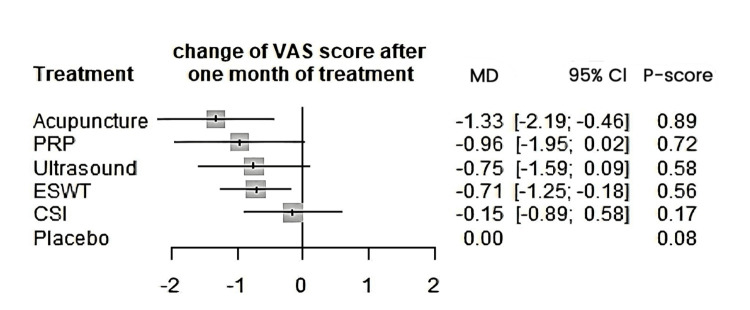
Forest plot comparing the mean difference of all interventions with placebo for change of VAS score after one month. ESWT: Extracorporeal shock wave therapy; PRP: Platelet-rich plasma; CSI: Corticosteroid injection; VAS: Visual analog scale; MD: Mean deviation; CL: Confidence interval

**Figure 5:**
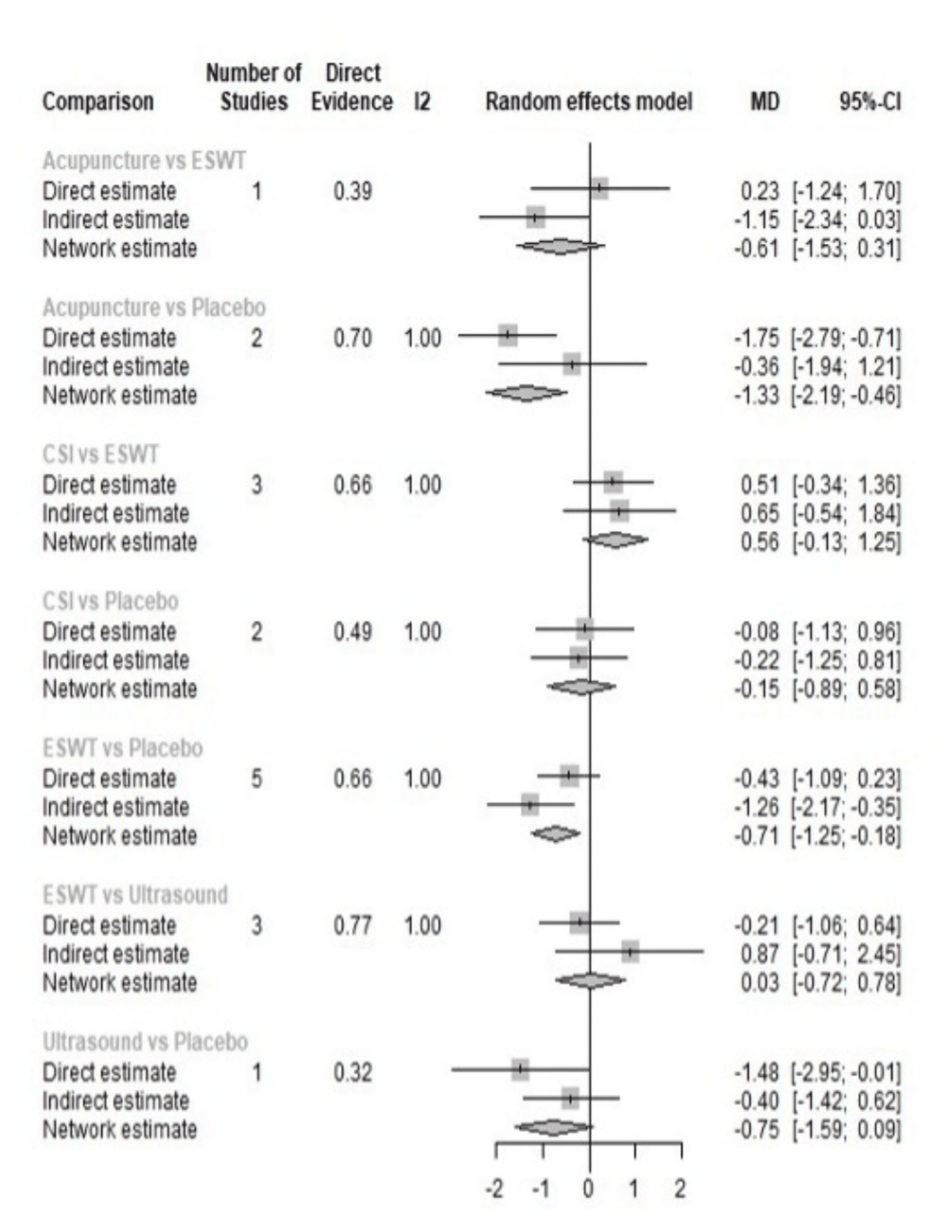
Forest plot showing the direct and indirect comparisons between interventions for change of VAS score after one month. ESWT: Extracorporeal shock wave therapy; CSI: Corticosteroid injection; VAS: Visual analog scale; MD: Mean deviation; CL: Confidence interval

**Figure 6:**
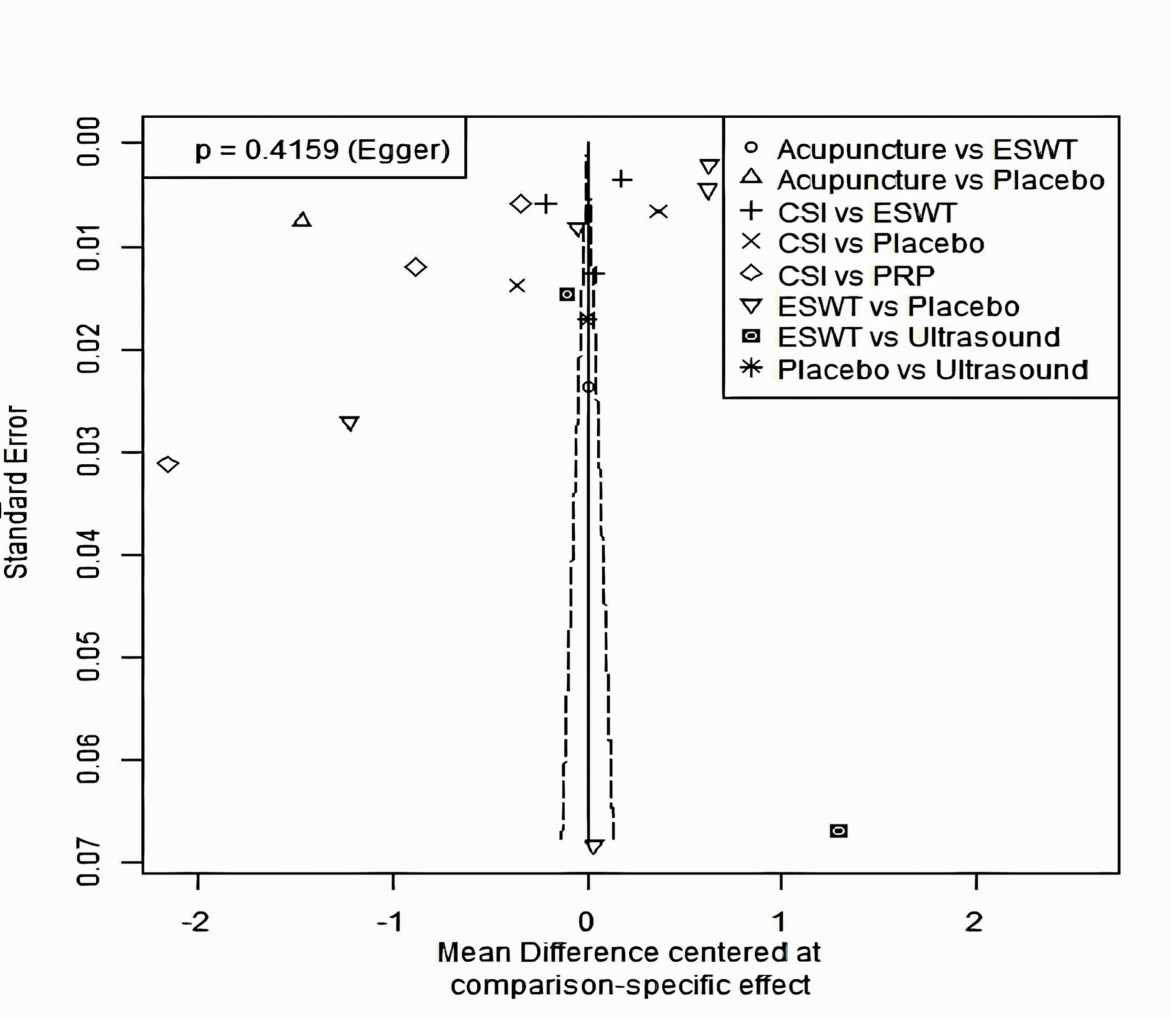
Funnel plot for change of VAS score after one month ESWT: Extracorporeal shock wave therapy; CSI: Corticosteroid injection; VAS: Visual analog scale

A total of 20 studies were included in the analysis of change in VAS score between six different treatment modalities (acupuncture, CSI, ESWT, PRP injection, USG therapy, and placebo). The most common comparison was between ESWT and placebo (Figure 2). The net heat plot showed significant heterogeneity when using a fixed-effect model, which was reduced when a random-effects model was used (Figure 3). The results of the network meta-analysis showed that acupuncture was associated with the highest reduction in pain compared to placebo, with an MD of -1.33 (95% confidence interval (95% CI) = -2.19 to -0.46) compared to placebo. All other treatments also showed moderate reduction in pain when compared to placebo, with CSI showing the least reduction in pain with an MD of -0.15 (95% CI = -0.89 to 0.58), as seen in Figure 4. Despite an insignificant MD between all treatment arms and placebo, the direct and indirect comparisons showed deviation from the null effect, indicating pain reduction when compared to placebo, as seen in Figure 5. Figure 6 further illustrates these direct and indirect effects, and all possible comparisons are shown in Table 3. Moreover, the funnel plot showed no significant publication bias, as Egger’s test resulted in a p-value of 0.42 (Figure 6).

**Table 3:** Matrix of comparisons for change of VAS score after one month ESWT: Extracorporeal shock wave therapy; PRP: Platelet-rich plasma; CSI: Corticosteroid injection; VAS: Visual analog scale

S. No.	V1	V2	V3	V4	V5	V6
1	Acupuncture	-	0.23 (-1.24; 1.70)	-1.75 (-2.79; -0.71)	-	-
2	-1.17 (-2.26; -0.09)	CSI	0.51 (-0.34; 1.36)	-0.08 (-1.13; 0.96)	0.81 (0.15; 1.47)	-
3	-0.61 (-1.53; 0.31)	0.56 (-0.13; 1.25)	ESWT	-0.43 (-1.09; 0.23)	-	-0.21 (-1.06; 0.64)
4	-1.33 (-2.19; -0.46)	-0.15 (-0.89; 0.58)	-0.71 (-1.25; -0.18)	Placebo	-	1.48 (0.01; 2.95)
5	-0.36 (-1.63; 0.91)	0.81 (0.15; 1.47)	0.25 (-0.70; 1.21)	0.96 (-0.02; 1.95)	PRP	-
6	-0.58 (-1.73; 0.57)	0.59 (-0.40; 1.58)	0.03 (-0.72; 0.78)	0.75 (-0.09; 1.59)	-0.22 (-1.41; 0.97)	Ultrasound

Change in VAS Score After Three Months

**Figure 7:**
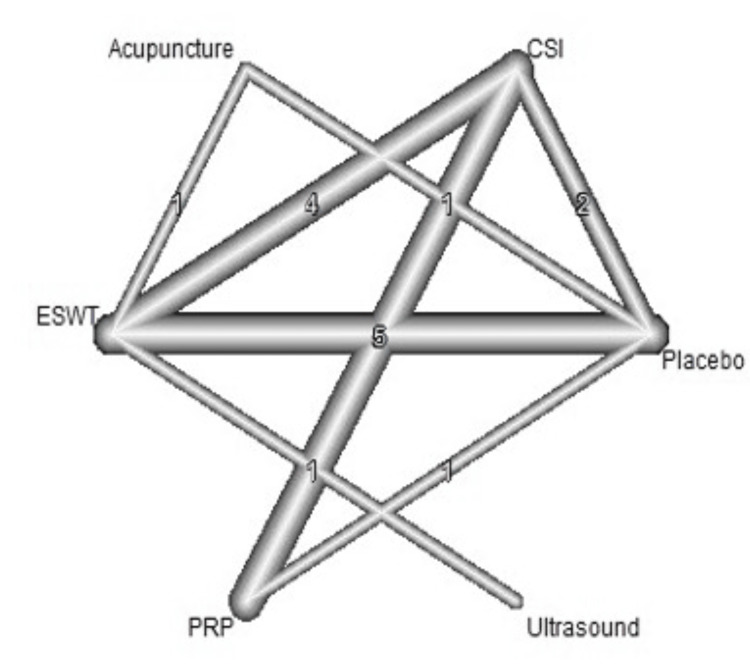
Network graph showing the connections between interventions and number of studies for each comparison for change of VAS score after three months. ESWT: Extracorporeal shock wave therapy; PRP: Platelet-rich plasma; CSI: Corticosteroid injection; VAS: Visual analog scale

**Figure 8:**
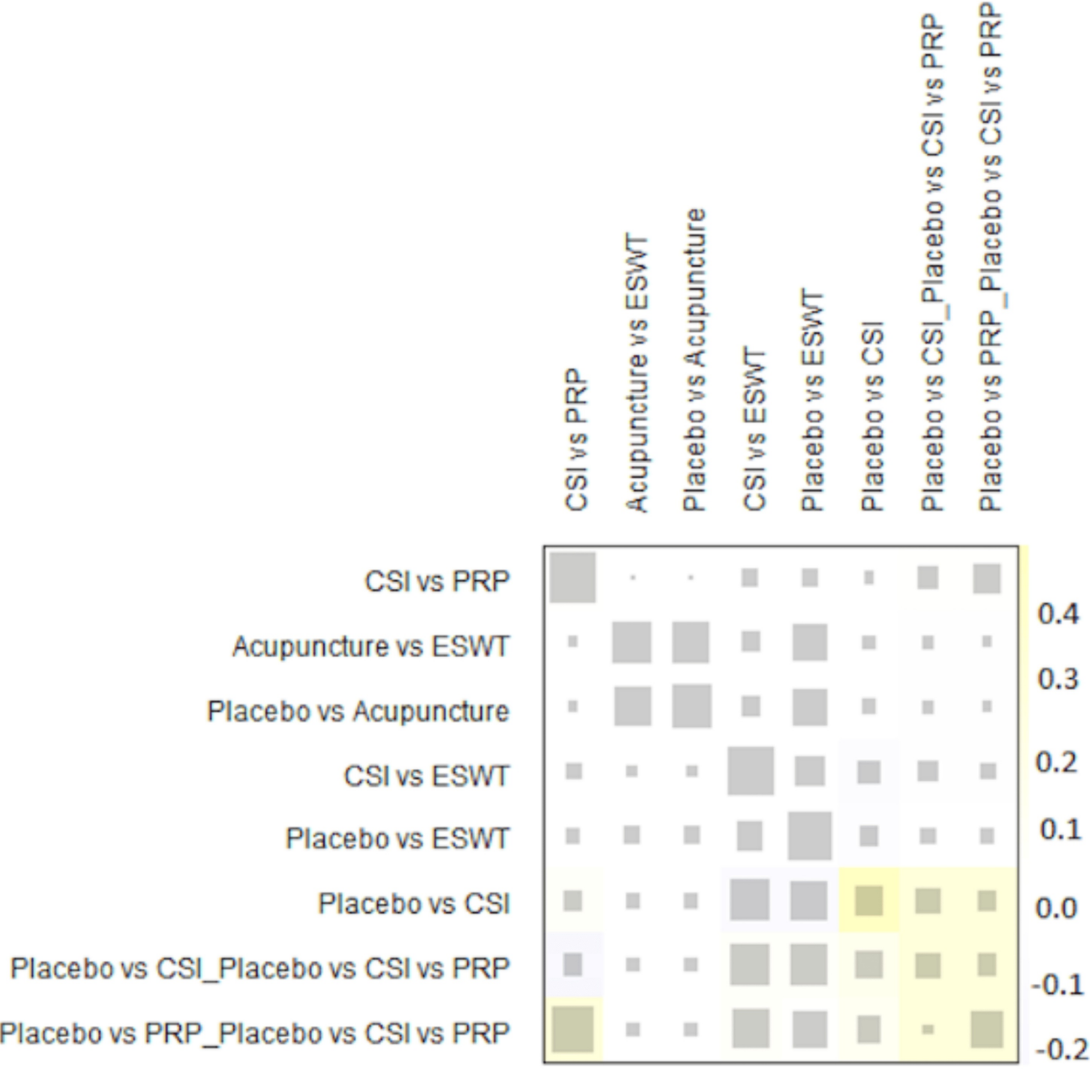
Net heat plot showing the consistency of the network for change of VAS score after three months. ESWT: Extracorporeal shock wave therapy; PRP: Platelet-rich plasma; CSI: Corticosteroid injection; VAS: Visual analog scale

**Figure 9:**
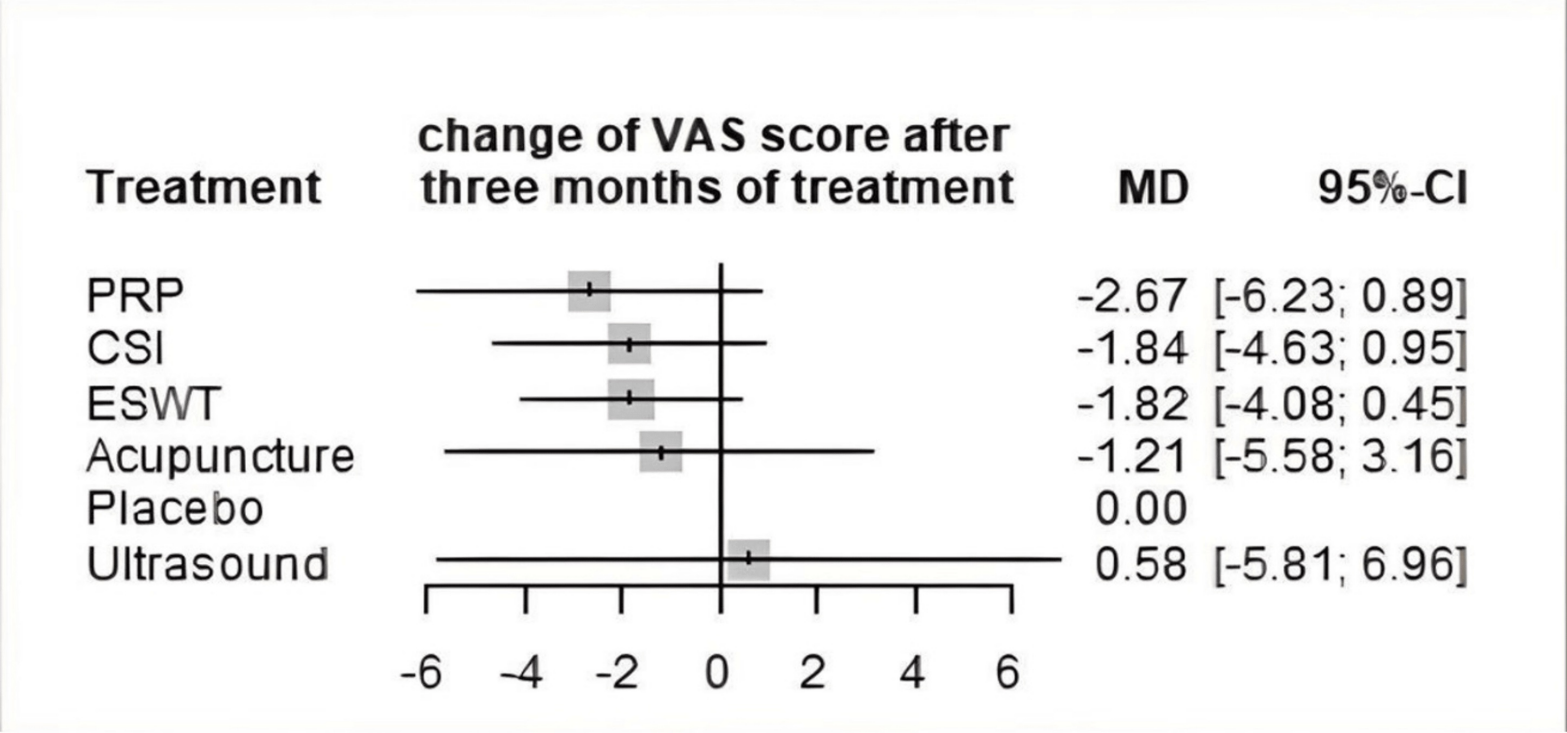
Forest plot comparing the mean difference of all interventions with placebo for change of VAS score after three months. ESWT: Extracorporeal shock wave therapy; PRP: Platelet-rich plasma; CSI: Corticosteroid injection; VAS: Visual analog scale; MD: Mean deviation; CL: Confidence interval

**Figure 10:**
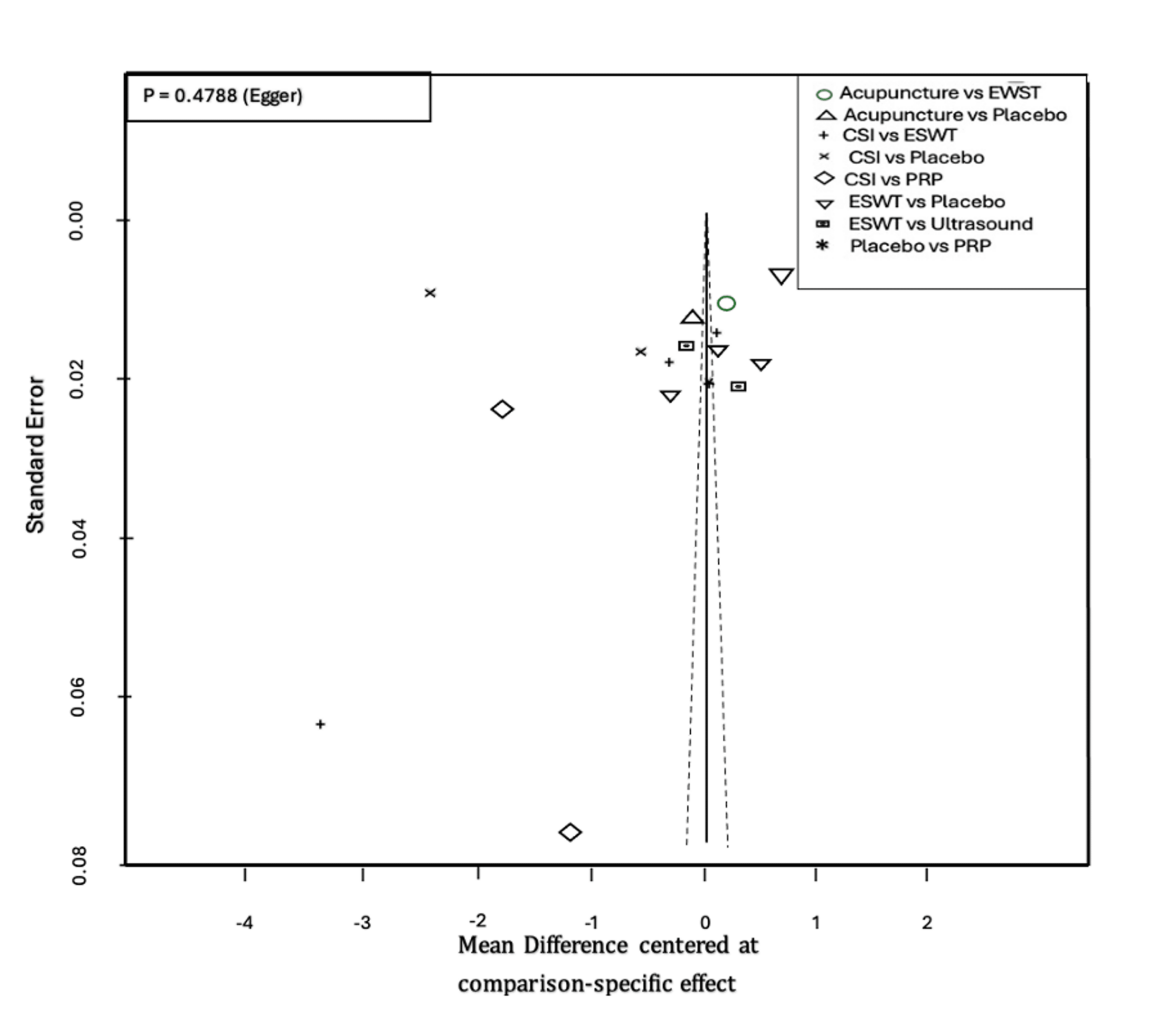
Funnel plot for change of VAS score after three months ESWT: Extracorporeal shock wave therapy; PRP: Platelet-rich plasma; CSI: Corticosteroid injection; VAS: Visual analog scale

**Figure 11:**
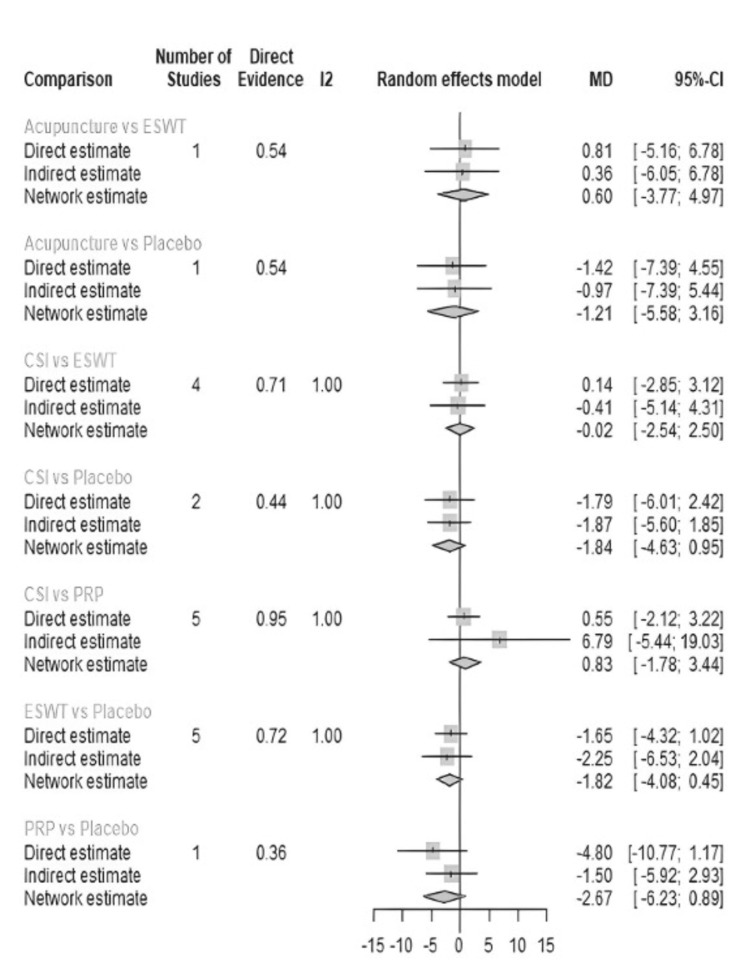
Forest plot showing the direct and indirect comparisons between interventions for change of VAS score after three months. ESWT: Extracorporeal shock wave therapy; PRP: Platelet-rich plasma; CSI: Corticosteroid injection; VAS: Visual analog scale; MD: Mean deviation; CL: Confidence interval

VAS score was measured at three months in 18 studies (Table 4). The most common comparisons were between ESWT against placebo and CSI with five and four studies, respectively (Figure 7). Similar to the one-month analysis, a random effects model was used to calculate the effect consistency of the network change of VAS score at three months using a net heat plot (Figure 8). The results of a Forrest plot comparing the MDs of treatments showed that the highest reduction in pain levels and effect size was achieved by PRP injection, with an MD of -2.67 (95% CI = -6.23 to 0.89; p = 0.77) compared to placebo, followed by CSI, with an MD of -1.84 (95% CI = -4.63 to 0.95; p = 0.61), and finally ESWT, acupuncture, and USG therapy (Figure 9). The node-splitting plot shown in Figure 9 indicates a consistent network via Egger’s test (p = 0.48), indicating no statistically significant publication bias (Figure 10). When treatment arms were compared with each other, the confidence interval of net effects of all treatments showed no or minimal deviation from the null effect, indicating no superiority between treatments (Figure 11).

**Table 4:** Matrix of comparisons for change of VAS score after three months ESWT: Extracorporeal shock wave therapy; PRP: Platelet-rich plasma; CSI: Corticosteroid injection; VAS: Visual analog scale

S. No.	V1	V2	V3	V4	V5	V6
1	Acupuncture	-	0.81 (-5.16; 6.78)	-1.42 (-7.39; 4.55)	-	-
2	0.62 (-4.23; 5.48)	CSI	0.14 (-2.85; 3.12)	-1.79 (-6.01; 2.42)	0.55 (-2.12; 3.22)	-
3	0.60 (-3.77; 4.97)	-0.02 (-2.54; 2.50)	ESWT	-1.65 (-4.32; 1.02)	-	-2.39 (-8.36; 3.57)
4	-1.21 (-5.58; 3.16)	-1.84 (-4.63; 0.95)	-1.82 (-4.08; 0.45)	Placebo	4.80 (-1.17; 10.77)	-
5	1.46 (-3.92; 6.83)	0.83 (-1.78; 3.44)	0.85 (-2.62; 4.32)	2.67 (-0.89; 6.23)	PRP	-
6	-1.79 (-9.18; 5.61)	-2.41 (-8.89; 4.06)	-2.39 (-8.36; 3.57)	-0.58 (-6.96; 5.81)	-3.25 (-10.15; 3.66)	Ultrasound

Efficacy of Outcomes

In terms of efficacy for short-term VAS reduction, the acupuncture treatment arm had a better effect on VAS score reduction compared to the placebo and other treatment arms at the one-month mark. Acupuncture ranked highest in the forest plot in terms of MD, followed by PRP injection, USG therapy, ESWT, and CSI. However, at the three-month mark, acupuncture treatment was ranked below PRP injection, CSI, and ESWT, with PRP injection ranking the highest. There were no significant differences between treatments when compared against each other (Figure 11).

Discussions

Acupuncture Intervention

In TCM theory, plantar fasciitis is classified as part of heel pain diagnosis in the syndrome differentiation category. According to TCM, heel pain is due to the stagnation of qi and blood in certain channels or due to kidney deficiency. Repetitive stress and trauma to the heel due to inappropriate movement will cause stagnation of qi and blood in the channels, resulting in pain [56]. The kidney dominates bones, and the kidney meridian flows to the heel covering most of the sole. When the kidney becomes weak, the heel will lack nourishment from the kidney meridian, resulting in injury or wear and tear [57]. The acupuncturist decides on needling points depending on the cause of the heel pain and related meridian(s). Once decided, the needling is done by either the reinforcing or reducing technique depending on the cause of heel pain. Use of the reinforcing technique is more common, as heel pain is related to kidney deficiency or due to stagnation of qi and blood. This technique can be done either manually with an acupuncture needle or with the help of electro-acupuncture. When relating acupuncture to modern medicine to manage pain, numerous mechanisms have been described, including central opioid pain inhibition [57], the anti-inflammation effect [58], and diffuse noxious inhibitory control (DNIC) [59]. Needle insertion may alleviate pain by central opioid pain inhibition or DNIC, and provide an anti-inflammatory effect. However, this mechanism cannot be related directly to the specific sites where the needle is inserted, which varies based on diagnosis and pathology.

The current literature and studies suggest that acupuncture is an effective mode of treatment for plantar fasciitis [60]. One of the earliest English-language studies was published by Tillu and Gupta in 1998. They reviewed 18 patients who had failed standard first-line treatment and were treated with acupuncture intervention for six weeks and found a significant reduction in VAS pain score [61]. In an RCT by Kumnerddee and Pattapong on the efficacy of electro-acupuncture in chronic plantar fasciitis, 30 patients who failed conservative treatment for at least six weeks were selected [41]. The control group received five weeks of standard treatment, and the acupuncture group received five weeks of acupuncture sessions at least twice a week. After these patients were followed up for six weeks, 80% reported successful pain control. Similarly, a 2018 systematic review by Clark and Tighe on the effectiveness of acupuncture in plantar heel pain also concluded that this mode of treatment was effective and comparable to other treatments such as stretching and the use of splints [62].

*Analysis* *Method*

A network meta-analysis is a complex statistical analysis comparing the effectiveness of several treatment arms directly and indirectly, which is especially important when comparing treatment arms for a condition where there is no definitive management or one single effective treatment. Combining both direct and indirect evidence can estimate the relative effect of each treatment, and by including indirect evidence, there is an increase in the estimated effect size. Network meta-analysis has several important benefits. First, it allows available information to be extracted from several sets of studies into one analysis. Second, it can incorporate indirect evidence in a network, which cannot be done using conventional network meta-analysis. Finally, assuming all assumptions are met, and the results conclusive, network meta-analysis can determine the type of treatment preferable for the target population [63,64].

Network meta-analysis is sometimes criticized because of its use of indirect evidence in cases where evidence for direct comparison is available. The important point in network meta-analysis is the participants in randomized trials were randomly allocated to one treatment arm, treatment A or treatment B, but the trial condition was not selected using this network method [65].

The use of a frequentist framework is still more common than a Bayesian framework, as the results of the former are more easily understood. Frequentist focuses on direct comparisons between treatments and combines them using a random effects model. It also uses indirect comparisons, but interpretation in a frequentist framework uses p-values and CIs. In the Bayesian approach, instead of just point estimates, it provides full posterior distributions for each treatment effect, allowing for more nuanced comparisons. For example, it can give the probability that one treatment is the best among all, which is not directly available from a frequentist analysis. However, the disadvantage of using this model is that it does not support meta-regression. Frequentism uses a theoretical approach to interpret the probability of an event; when the process is repeated multiple times, the probability of the event in terms of how often it is expected to occur is established [66,67].

The frequentist method uses a p-score to rank treatment, which is equivalent to the surface under the cumulative ranking analysis (SUCRA) score used in the Bayesian method. In this method, when the MD shows a negative value or effect size, this indicates that the treatment is effective [66-68].

This network meta-analysis study did have some limitations. We only used the VAS score as a pain parameter, as this is the most frequently used parameter in most studies; we were unable to use foot function parameters (e.g., foot function index), as few studies used them. In addition, many studies were excluded from our meta-analysis because they failed to meet the inclusion criteria, limiting the total number of included studies.

Acupuncture as a Viable Treatment Option

As this network meta-analysis was mainly used to compare the use of acupuncture as an alternative to other non-surgical treatment options, we faced various challenges in finding high-quality studies to include. Studies involving acupuncture are seldom published in mainstream or English-language journals. Even with the use of the CKNI database, we were only able to include one study with an average-sized sample. Studies involving acupuncture treatment are impossible to do double-blinded, which affects their quality. Another problem we faced was the complexity and nuances of acupuncture treatment. In a condition like plantar fasciitis, syndrome differentiation is variable according to TCM. Therefore, the point of treatment selected for each patient was individualized according to the syndrome and not able to be synchronized among all patients. Although certain studies mention the use of certain points on all patients, this approach is potentially debatable.

The initial proposal to study the long-term effect of acupuncture was unable to be met due to the unavailability of long-term studies, which limited our analysis to the short-term effects of acupuncture treatment and other modalities such as ESWT, USG therapy, CSI, and PRP injection. Although long-term results would be preferable to assist clinicians in decision-making, this study could only conclude the effectiveness of acupuncture in the short term.

Many of the above-mentioned treatments were compared as a single treatment modality and not in combination with other forms of treatment. However, in reality, a combination treatment is most likely to be performed. For example, patients can be prescribed ESWT weekly in combination with acupuncture treatment or PRP injection. As a combination treatment model usually leads to better outcomes, we suggest that more studies using combination treatment be performed and published.

One drawback of acupuncture treatment is that it is not readily available in most modern hospitals. In addition, the learning curve to master an acupuncture treatment is steep and requires years of knowledge and clinical practice compared to treatment options such as CSI or PRP injection, which can be learned in one setting. In contrast, EWST and USG treatment options are readily available in most hospitals and usually performed by a trained physiotherapist.

## Conclusions

Acupuncture treatment for plantar fasciitis is effective in short-term management and should be prescribed by well-trained clinicians. It should be considered a second-line treatment for plantar fasciitis together with other common treatment options such as ESWT, PRP injection, CSI, and USG therapy. Further long-term studies should be done to effectively determine the long-term outcomes of acupuncture treatment.
